# Additive Engineering to Grow Micron‐Sized Grains for Stable High Efficiency Perovskite Solar Cells

**DOI:** 10.1002/advs.201901241

**Published:** 2019-07-26

**Authors:** Hua Li, Guohua Wu, Wanyi Li, Yaohong Zhang, Zhike Liu, Dapeng Wang, Shengzhong (Frank) Liu

**Affiliations:** ^1^ Key Laboratory of Applied Surface and Colloid Chemistry Ministry of Education Key Laboratory for Advanced Energy Devices Shaanxi Engineering Lab for Advanced Energy Technology School of Materials Science and Engineering Shaanxi Normal University Xi'an 710119 China; ^2^ Faculty of Informatics and Engineering The University of Electro‐Communications 1‐5‐1 Chofugaoka Chofu Tokyo 182‐8585 Japan

**Keywords:** additive engineering, hydrophobic, micron‐sized grain, perovskite solar cells, stability

## Abstract

A high‐quality perovskite photoactive layer plays a crucial role in determining the device performance. An additive engineering strategy is introduced by utilizing different concentrations of N,1‐diiodoformamidine (DIFA) in the perovskite precursor solution to essentially achieve high‐quality monolayer‐like perovskite films with enhanced crystallinity, hydrophobic property, smooth surface, and grain size up to nearly 3 µm, leading to significantly reduced grain boundaries, trap densities, and thus diminished hysteresis in the resultant perovskite solar cells (PSCs). The optimized devices with 2% DIFA additive show the best device performance with a significantly enhanced power conversion efficiency (PCE) of 21.22%, as compared to the control devices with the highest PCE of 19.07%. 2% DIFA modified devices show better stability than the control ones. Overall, the introduction of DIFA additive is demonstrated to be a facile approach to obtain high‐efficiency, hysteresis‐less, and simultaneously stable PSCs.

## 1 Introduction

In the past few years, the development of organic–inorganic hybrid perovskite solar cells (PSCs) has progressed rapidly. A power conversion efficiency (PCE) of PSCs has sharply increased from the initial 3.8% in 2009 to over 24% in 2019,^1^, ^2^, ^3^ achieving comparable photovoltaic performance compared to commercial silicon‐based solar cells. Other advantages of thin‐film PSCs including low‐cost solution process and facile fabrication process extremely impel PSCs candidates for photovoltaic applications.

Currently, the two critical issues that prevent the large‐scale commercialization of PSCs are the current–voltage (*I–V*) hysteresis and the inherent instability of the perovskite film.^4^ The *I–V* hysteresis in PSCs is related to the unbalanced photoexcited electrons and holes, ferroelectric polarization, ion migration, charge carrier trapping, transient capacitive current, and so on.^5^, ^6^ All of these factors are strongly associated with the quality of the perovskite films. It has been demonstrated that high‐quality perovskite thin films play a vital role in obtaining high‐performance PSCs with excellent stability since the surface morphology and crystallization behavior of perovskite thin films can significantly affect the photoelectric characteristics, such as the charge carrier mobility, charge separation and collection efficiencies, recombination mechanics, lifetime, and diffusion‐length of perovskite thin films.^7^, ^8^, ^9^, ^10^, ^11^ Thus, it would be highly demanded to obtain high‐efficient, hysteresis‐less even hysteresis‐free, and simultaneously stable PSCs by developing an effective methodology for precise perovskite film amelioration.

The addition of a tiny amount of chemical additives in the perovskite precursor solution has been widely demonstrated as one of the most effective strategies to control the crystallization process,^12^, ^13^ like grain size or morphology of perovskite thin film, thus enhancing the PCE of the PSC devices. Recently, more and more additives for PSCs emerged. A commonly utilized PbI_2_ and *N*,*N*‐dimethyl sulfoxide (DMSO) as effective self‐doping additives in the precursor solution were initially proposed to control the grain size and crystalline of perovskite films, contributing to a best‐performing PCE of 20.2% due to a compact uniform morphology with larger grain size.^14^, ^15^ Alkyl halide additives with different alkyl chain lengths and end groups in mixed halide perovskites (MAPbI*_x_*Cl_3−_ *_x_* with MA = CH_3_NH_3_) show the enhanced crystallization of perovskite thin films and significantly enhanced device performance.^16^ Later, the perovskite crystal growth can be accelerated by the addition of a small amount of zwitterionic sulfamic acid from irregular 100 nm to regular and square 500 nm, which facilitates electron transfer process.^17^ Jiang and co‐workers incorporated a bifunctional hydroxylamine hydrochloride into pristine perovskite solution to enhance the quality of perovskite with larger grain size (from 150 to 350 nm) and lower defect density, thus enhancing the efficiency from 16.85% to 18.69% as well as their thermal and air stabilities.^18^ By a two‐step sequential ethyl acetate interfacial processing, Fei and co‐workers employed thiourea as additive in the perovskite precursor to effectively control the nucleation and subsequent crystal growth processes presenting compact microsized and monolithically grained perovskite films.^19^ The resulting PSCs demonstrate an impressive PCE of 18.46% and excellent stability in ambient air. Recently, Cai et al.^20^ introduced trimethylammonium chloride (TACl) as additive to prepare perovskite films. The addition of TACl increases device efficiency from 19.1% to 20.9% and the average grain size from 350 to 500 nm. The larger grained films often exhibit longer excited‐state lifetime, lower trap density, and higher PCE with relatively less open‐circuit voltage (*V*_oc_) loss.^21^, ^22^, ^23^ Although the above mentioned additive can achieve large‐grained perovskite films, unfortunately, fabricating perovskite films with much more larger grain sizes along with lower trap densities using a novel additive is still a new challenge.

**Figure 1:**
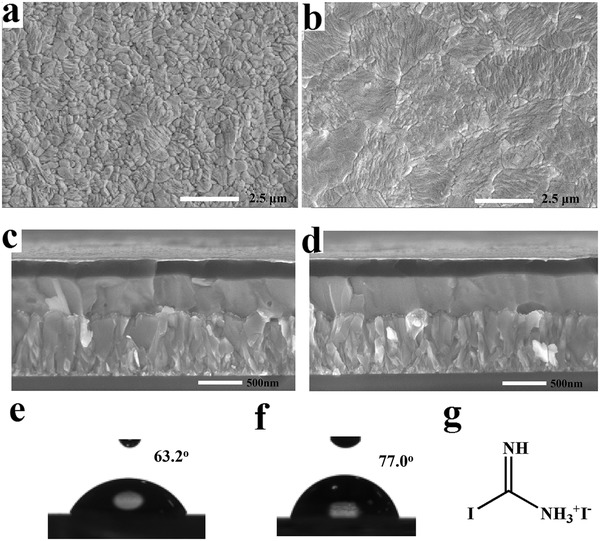
Surface‐view scanning electron microscopy (SEM) images of perovskite films a) without and b) with 2% DIFA additive. Cross‐sectional SEM images of planar PSC structure c) without and d) with 2% DIFA additive. The water contact angle data of perovskite films e) without and f) with 2% DIFA additive. g) The molecular structure of DIFA.

In this study, we introduce a novel small molecule N,1‐diiodoformamidine (DIFA, **Figure** **1**g) additive into the FA_0.85_MA_0.15_PbI_3_ (FA = HC(NH_2_)_2_) perovskite precursor solution prior to spin‐coating approach for the fabrication of perovskite thin films. As an organic amidine compound, DIFA endowed with ammonium cation and C–I group possesses the structure resulted from C–I instead of C–H in formamidinium iodide (FAI). The incorporation of hydrophobic C–I group can significantly improve the molecular hydrophobicity of the perovskite film, which effectively protects the perovskite films against water penetration and ultimately enhances the PSC humidity stability. The grain size of bication perovskite film can be significantly modulated from nanoscale to microscale (nearly 3 µm) upon varying the amounts of DIFA, which thereby decreases the trap density in the film. In this paper, it is identified that the addition of DIFA in the perovskite precursor solution is an extremely effective method to significantly enhance the PSC photovoltaic performance and its stability. 2% DIFA‐modified PSCs demonstrate a champion PCE of 21.22% with a high short‐circuit current (*J*_sc_) of 25.05 mA cm^−2^, *V*_oc_ of 1.10 V, and fill factor (FF) of 77.3%. This efficient FA_0.85_MA_0.15_DIFA_0.02_PbI_3_‐based device performance paves the way for the commercialization of PSCs in the future.

## 2 Result and Discussion

As depicted in the high‐resolution SEM images, a relatively compact and smooth surface morphology of the perovskite films without and with 2% DIFA additive is presented in Figure 1a,b. The SEM images of perovskite films with the other concentrations of DIFA additive and the corresponding distribution of perovskite grain size are illustrated in Figures S1 and S2 (Supporting Information). The grain size distribution of the pristine perovskite film ranges from 200 to 1000 nm with the peak ≈500 nm. By adding 2% DIFA, the average crystal grain size significantly increases and becomes more uniform ranging from 800 to 2900 nm with the peak ≈1600 nm, which is consistent with the cross‐sectional SEM images of planar PSC structure without and with 2% DIFA additive as shown in Figure 1c,d. However, when the content of DIFA increases above 3%, the average perovskite crystal grain size becomes smaller again and shows the smallest appear at 8%. It is believed that the excess of DIFA accelerated the formation of disordered sol–gel precursor phase during the early stage of the spin‐coating process, which will suppress the charge transfer and collection in the PSC devices.^24^, ^25^ In order to better understand the interaction of DIFA with perovskite, ^1^H NMR measurement of a deuterated dimethylsulfoxide (DMSO) perovskite solution with the same concentration as the perovskite precursor solution in PSC fabrication process without or with 2% DIFA is performed as shown in Figures S3 and S4 (Supporting Information). It is found that in the pristine perovskite solution, the two resonance single peaks of HC(NH_2_)_2_^+^ are at 8.56 and 7.79 ppm, respectively. Upon addition of 2% DIFA to the perovskite solution, the original peak at 8.56 ppm splits into two new peaks at 8.88 and 8.56 ppm. At the same time, the original single peak at 7.79 ppm splits into multiple peaks due to interaction between cationic HC(NH_2_)_2_^+^ and DIFA. Taking into account this supramolecular interaction between the DIFA molecules and HC(NH_2_)_2_^+^,^26^, ^27^ it can be inferred that the addition of 2% DIFA can delay the crystallization rate of perovskites and facilitate the growth of high‐quality perovskite films with less grain boundaries, which is favorable for boosting the resulting solar cell performance.^24^, ^25^ Compared with the resonance single peak of MAPbI_3_ ≈7.4 ppm as shown in Figure S5 (Supporting Information), there is nearly no split and shift but broadening upon the addition of 2% DIFA indicating possible no interaction between the DIFA molecules and CH_3_NH_3_^+^_._ The water contact angle test of the perovskite films with different concentration of DIFA additive is performed as shown in Figure S6 (Supporting Information) and the average data are listed in Table S1 (Supporting Information). Compared with the pristine perovskite film with a smaller contact angle of 63.2° (Figure 1e), the water contact angle can be increased to 73.3°, 77° (Figure 1f), 78.3°, 79.2°, and 80.5° by introducing 1%, 2%, 3%, 6%, and 8% DIFA, respectively, which indicates that the effect of relatively hydrophobic C–I group in view of the amphiphilic nature of DIFA. Figure S7 (Supporting Information) shows the 3D atomic force microscopy (AFM) height images of perovskite films on TiO_2_ layer without or with 2% DIFA. The root‐mean‐square (RMS) roughness is 19.5 nm for pristine perovskite film and 16.9 nm for 2% DIFA‐modified perovskite film, respectively. This indicates that 2% DIFA can facilitate the flatness of the perovskite films. We have also performed the conductivity measurement with the Au/perovskite/Au device as shown in Figure S8 (Supporting Information). By the addition of 2% DIFA, the conductivity of bication perovskite film can increase from 7.12 × 10^−3^ to 9.89 × 10^−3^ S cm^−1^ indicating superior charge transport induced by the less grain boundaries in the perovskite film.

**Figure 2:**
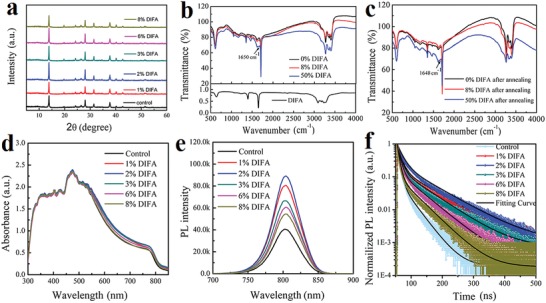
a) XRD patterns of fresh perovskite films, b) Fourier transform infrared (FT‐IR) spectra of perovskite films and DIFA solid, c) the perovskite films after annealing, d) absorption spectra, e) photoluminescence spectra, and f) transient‐state photoluminescence spectra of perovskite films incorporating different concentrations of DIFA.

In addition, the effect of DIFA additive to the crystallinity of perovskite films is clearly proved from the X‐ray diffraction (XRD) patterns as shown in **Figure** **2**. It can be seen that no significant shift of the diffraction peaks is observed after the addition of DIFA, which infers that the addition of DIFA did not penetrate into the pristine FA_0.85_MA_0.15_PbI_3_ perovskite crystal lattice. The full width at half maxima (FWHM) of the characteristic peaks in the FAMA perovskite films with the increasing concentration of DIFA additive shows an obvious decrease first and then increase with the smallest appear at 2%, which certifies that the addition of 2% DIFA presents the best perovskite crystallinity quality. Overall, the addition of DIFA can contribute the perovskite crystallinity process. The FT‐IR spectra of the perovskite films with different concentrations of DIFA before and after annealing are presented in Figure 2b,c. As shown in Figure 2b, the feature band at 1650 cm^−1^ can be assigned to the vibration of C=N group in DIFA molecule. The broad region of relatively strong peaks between 3080 and 3300 cm^−1^ can be assigned to the vibration of amine units. In our study, tiny amount of DIFA additive is introduced in the perovskite precursor solution while no change of stretching vibration is observed. By further increasing DIFA content to 50%, the feature band at 1650 cm^−1^ is clearly appeared, which is assigned to the vibration of C=N group in DIFA. After annealing at 150 °C for 30 min, this feature band shows a little lower wavenumber shift to 1648 cm^−1^. The elements of N, I, and Pb of perovskite are tracked from point A and point B at the grain boundary by energy‐dispersive X‐ray spectrometry (EDX) as shown in Figure S9 (Supporting Information). It is found that the element ratio of Pb to I is 3, which is close to the theoretical value of FA_0.85_MA_0.15_PbI_3_ perovskite. Then it is significantly increasing to 3.4 with the addition of 2% DIFA, which indicates that DIFA should be also effective and remained at the perovskite grain boundaries. The absorption spectra of FA_0.85_MA_0.15_PbI_3_ perovskite films with different concentrations of DIFA additive are depicted in Figure 2d. The presence of DIFA additive does not affect the band edge of absorption besides the absorption intensity. As shown in Figure 2e, all the steady‐state PL spectra of perovskite films exhibit a strong emission peak at the positions similar to their absorption edges revealed in UV–vis spectra. In comparison with the pristine perovskite film, the enhanced PL intensity of film with DIFA indicates that the addition of DIFA can reduce the defect states in those perovskite films.^28^, ^29^ To accurately evaluate the lifetime of the charge carriers and the density of defect state in the perovskite films, the measurements of time‐resolved PL spectra (Figure 2f) are performed.^30^ The average lifetimes of FA_0.85_MA_0.15_PbI_3_ perovskite films based on different concentrations of DIFA (0, 1%, 2%, 3%, 6%, 8%) are 15, 50, 55, 43, 33, and 25 ns, respectively (see Table S2, Supporting Information). These results further verify that a proper addition of DIFA plays a key role in decreasing the defects in the perovskite films and simultaneously prolonging the carrier lifetime of the perovskite films.

**Figure 3:**
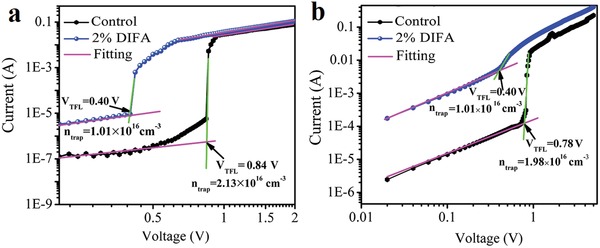
Dark current–voltage characteristics of a) electron only devices and b) hole only devices based on perovskite film with 0% and 2% DIFA.

Then the density analysis of defect states in perovskite films is carried out through FTO/TiO_2_/FA_0.85_MA_0.15_PbI_3_/PCBM/Ag (electron only) or FTO/PEDOT:PSS/FA_0.85_MA_0.15_PbI_3_/spiro‐OMeTAD/Au (hole only) device to accurately identify the defect density variation of DIFA. The typical dark current density–voltage characteristics as shown in **Figure** **3** can be divided into three regions: Ohmic linear relation at the low bias voltage, a rapid nonlinear trap‐filled limit (TFL) region, and the space‐charge‐limited‐current region.^20^ The TFL voltage (*V*_TFL_) can be extracted from the curves as the intersection between the Ohmic and TFL region. The concentrations of trap states can be calculated from the following equation

(1) $$V_{\text{TFL}}\;=\;\frac{{\text{en}}_{\text{trap}}L^{2}}{2\varepsilon \varepsilon_{0}}$$

where *L* is the thickness of the perovskite film, ε is relative dielectric constant of perovskite), and ε_0_ is the vacuum permittivity (8.854 × 10^−14^ F cm^−1^).^24^, ^31^ By the addition of 2% DIFA, the electron and hole trap densities in the perovskite films are calculated to be 1.01 × 10^16^ and 1.01 × 10^16^ cm^−3^, respectively, which are much lower than the corresponding values for the control device (2.13 × 10^16^ cm^−3^ for electron only device and 1.98 × 10^16^ cm^−3^ for hole only device). This confirms that the addition of DIFA additive is conducive to lower the defect density in perovskite films, which is beneficial to eventually reduce hysteresis in the perovskite devices.^32^, ^33^, ^34^ As we all known that the origin of the interfacial defects in the perovskite film is halogen vacancy,^35^ we speculate that the moderate presence of I^−^ in DIFA can precisely fill this vacancy site inside the perovskite crystal and on the surface of perovskite film, which directly leads to a lower density of defects in the perovskite films by addition of 2% DIFA and hence the formation of larger and flatter grains. However, the excess DIFA simultaneously forms new trap centers increasing the electron trap density as demonstrated in Figure S10 (Supporting Information), which is not conductive to improving the device performance.

**Figure 4:**
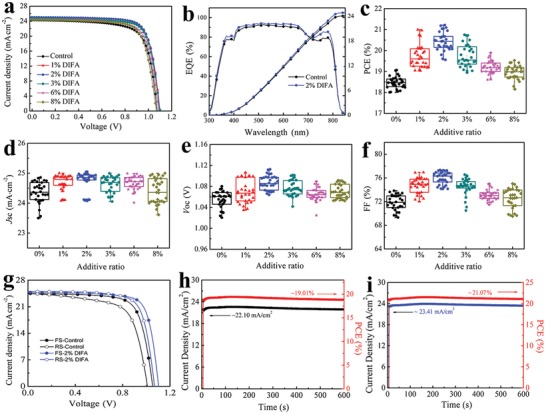
a) Current density–voltage (*J–V*) curve of the PSC devices with the different concentrations of additive from 0% to 8% under one Sun AM 1.5G (100 mW cm^−2^). b) EQE spectra with integrated current densities of the devices without and with 2% DIFA. c–f) Statistical distribution of the photovoltaic parameters for PSC devices with the different concentrations of additive from 0% to 8%: c) distribution of PCE; d) distribution of *J*_sc_; e) distribution of *V*_oc_; f) distribution of FF. g) *J–V* curves of PSCs with and without DIFA measured by forward (short circuit‐to‐open circuit) and reverse (open circuit‐to‐short circuit) scans with 0.02 V voltage step. Stable output curves of *J*_sc_ and PCE of PSCs without h) and i) with 2% DIFA addition for 600 s.

The influence of DIFA additive on the planar heterojunction PSC device performance is then investigated and the PSC structure we fabricated is of glass/FTO/TiO_2_/FA_0.85_MA_0.15_PbI_3_/spiro‐OMeTAD/Au as illustrated in Figure 1c. The best‐performing photovoltaic characteristics of the solar cell devices with various concentrations of DIFA additive are summarized in **Table** **1**. The typical *J–V* curves of these PSCs are displayed in **Figure** **4**a and the PCE distribution for a batch of 30 perovskite solar cells is shown in Figure 4c–f presenting the superior reproducibility of the PSC devices. The control solar cell device without DIFA addition shows a maximum PCE of 19.07%, which is substantially improved to 21.22% when 2% of DIFA is added in the perovskite precursor solution, primarily improving *J*_sc_, *V*_oc_, and FF. This is mainly attributed to the high‐quality perovskite films upon 2% DIFA addition with reduced grain boundaries, higher conductivity, lower trap densities, and relatively smaller series resistance compared with the control device. In contrast, further adding the DIFA content from 2% to 8% deteriorates the solar cell performance including *J*_sc_, *V*_oc_, and FF due to the disruptive crystallinity of perovskite films, which is proved from the results of SEM and XRD measurements. Figure 4b shows the external quantum efficiency (EQE) spectra of the PSC devices without and with the optimized concentration of DIFA additive, which is close to the *J*_sc_ values obtained from above *J–V* curves. Moreover, both reverse (RS) and forward (FS) *J–V* scans are also investigated in order to study the hysteresis effects of the devices with various concentrations of DIFA additive as shown in Figure 4g. Here a hysteresis index (HI) is summarized in Table S4 (Supporting Information), which demonstrates the hysteresis degree in PSC device. As reported, the hysteresis is originated from the defects, the carrier trapping, and capacitive effects.^5^, ^36^ The control device with 18.91% for the reverse scan (RS) and 16.50% for the forward scan (FS) shows an obvious hysteresis giving a HI of 12.7. Interestingly, 2% DIFA PSC device presents a high PCE of 20.00% (FS) and 21.04% (RS) with a HI of 4.9. It can be seen that the relatively small deviations are measured in forward and reverse scans verified that the addition of DIFA is favorable to diminish the hysteresis in PSCs, which is ascribed to the reduced defects in the perovskite films with the addition of DIFA. The stable output curves of *J*_sc_ and PCE of PSCs without and with 2% DIFA addition at the maximum power condition are tested under light soaking in air as presented in Figure 4h,i. A stabilized photocurrent of 23.41 mA cm^−2^ is obtained for the PSC device with 2% DIFA corresponding to a stabilized PCE of 21.07%, which is higher than that of the pristine PSC device with a photocurrent of 22.10 mA cm^−2^ and a PCE of 19.01%. Therefore, a tiny amount of DIFA can effectively ameliorate the light‐soaking stability of PSC devices.

**Table 1:** Summary of the best‐performing photovoltaic parameters of the PSCs with different contents of DIFA

PSCs	*V*_oc_ [V]	*J*_sc_ [mA cm^−2^]	FF [%]	PCE [%]
Control	1.08	24.29	72.9	19.07
1% DIFA	1.10	24.79	76.9	20.99
2% DIFA	1.10	25.05	77.3	21.22
3% DIFA	1.10	24.69	76.3	20.75
6% DIFA	1.09	24.47	74.6	19.90
8% DIFA	1.07	24.36	75.0	19.53

**Figure 5:**
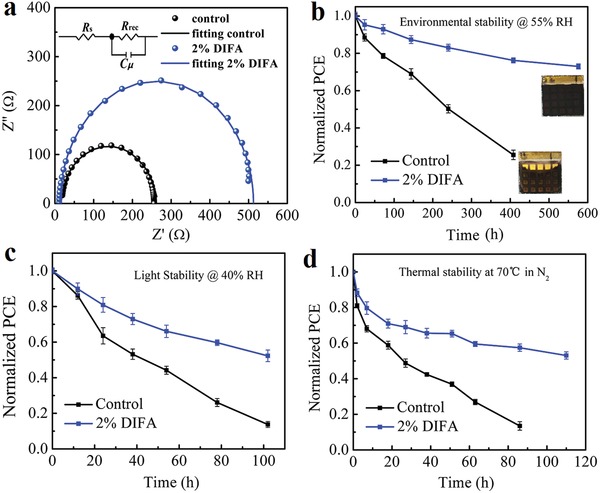
a) Nyquist plots of the PSCs without and with 2% DIFA addition under 1.05 V bias. b) The dark environmental stability of the PSCs without and with 2% DIFA addition with a relative humidity of ≈55%. c) Light stabilities of PSCs without and with 2% DIFA addition for 100 h in xenon lamp aging box with a relative humidity of ≈40%. d) Thermal stabilities of PSCs without and with 2% DIFA addition at 70 °C under N_2_ atmosphere.

Electrochemical impedance spectra (EIS) were carried out to investigate the charge transport process in PSC devices.^37^, ^38^, ^39^, ^40^, ^41^ The Nyquist plots of the PSC devices at 1.05 V under dark conditions are shown in **Figure** **5**a. Two main interfacial resistances including the series resistance (*R*_s_) and recombination resistance (*R*_rec_) in parallel with a chemical capacitance (*C_µ_*) at the TiO_2_/perovskite/HTL interface can be extracted from the EIS. The fitted *R*_s_ values are found to be in the order of control (18.00 Ω) >2% DIFA (12.60 Ω), which may cause the higher FF of the PSC device with 2% DIFA. This may be resulted from the reduced resistance of the passivated perovskite film, especially the addition of 2% DIFA. Compared with the control device (238 Ω), the higher fitted *R*_rec_ values are obtained as 500 Ω for 2% DIFA‐based devices indicating lower recombination rate at the TiO_2_/perovskite/HTL interface and resulting in better device performance with a higher *V*_oc_ parameter. Combined with the obtained *R*_rec_ and *C_µ_* according to a formula (τ_e_ = *R*_rec_ × *C_µ_*), the calculated carrier lifetimes (τ_e_) are 2.74 µs for the control PSC device and 4.49 µs for 2% DIFA‐based PSC device, respectively. The higher *R*_rec_ value as well as the longer carrier lifetime for 2% DIFA‐based PSC device reveals the more effective suppression of the charge recombination between perovskite grains and at the interface of the devices, which is favorable for improving the *V*_oc_ of PSCs. Furthermore, the long‐term stability investigation on PSC devices encapsulated in dark under ambient air conditions with an average temperature of 25 °C and humidity of 55% is displayed in Figure 5b. After a storage time of 576 h, the PSC device with 2% DIFA maintained over 73% of its original PCE while the control one drops its PCE quickly to about 25% after 408 h. It has been reported that the decomposition of the perovskite films is much easier to start from the surface vacancy sites.^42^, ^43^, ^44^ Due to the presence of vacancy sites in the perovskite films, water can strongly bind to the defective surface through the hydrogen bonds.^45^ The higher the quality of perovskite films, the less vacancy sites on the perovskite surface. The hydrophobic C–I group of DIFA can significantly improve the molecular hydrophobicity of the perovskite film, which effectively protects the perovskite films not penetrated by water and ultimately enhances the PSC humidity stability. The light stability of the PSC devices without and with 2% DIFA is also compared under one sun illumination without UV filter under the humidity of 40% as shown in Figure 5c. After a storage time of 100 h, the PCE of the control device degrades rapidly by 86% while the PSC device with 2% DIFA can maintain about 52% of its initial PCE, which indicates that the addition of DIFA can enhance the light stability of PSC device. The thermal stability of PSC devices without and with 2% DIFA is also investigated under N_2_ atmosphere as shown in Figure 5d. The control device only can maintain about 13% of its initial PCE after 86 h whereas PSC device with 2% DIFA can maintain above 57% of its initial PCE after 86 h and then gently degraded to 53% after 110 h. A significant degradation after the continuous light soaking or thermal condition is observed for the PSC device without or with 2% DIFA, which may be caused by the degraded perovskite active layer.^46^

## 3 Conclusion

A new additive coded as DIFA is introduced in the perovskite precursor to attain the high efficiency and hysteresis‐less PSCs. It has been found that the addition of DIFA can effectively control the crystallization process of the perovskite film, which can directly ameliorate crystallinity, enhance hydrophobic property, elongate the excited‐state lifetime, and diminish the trap densities in the perovskite films. By the addition of 2% DIFA, the compact, smooth, and large grained (nearly 3 µm) perovskite films are achieved resulted in the highly efficient defect passivation. Furthermore, a highest PCE of 21.22% is achieved for an active area of 0.09 cm^2^ modules with 2% DIFA additive. Compared with the control device, the PSC device based on 2% DIFA also exhibits an outstanding environmental, light, and thermal stabilities. Therefore, our rational additive approach has successfully obtained the high‐quality bication based perovskite films to afford highly efficient PSCs with high stability.

## 4 Experimental Section

*Synthesis of DIFA*: Cyanamide and HI were mixed with a 1:2.2 molar ratio and then stirred for 30 min at 70 °C under nitrogen atmosphere. The raw product was first collected by filtration and then purified by recrystallization. The white‐colored DIFA were collected, dried, and stored under argon atmosphere. ^1^H NMR (400 MHz, DMSO‐*d*_6_, δ/ppm): 7.77 (s, 4H); ^13^C NMR (100 MHz, DMSO‐*d*_6_, δ/ppm): 161.3; HRMS (ESI) *m*/*z*: [M‐I]^+^ calcd for CH_4_IN_2_, 170.9414; found, 170.9419. The NMR and mass spectra of DIFA are shown in Supporting Information.

*Device Fabrication*: A 2.5 × 2.5 cm^2^ piece of cleaning fluorine‐doped tinoxide (FTO) glass substrate was first treated under the UV−O_3_ plasma and then immersed in a 40 × 10^−3^ m TiCl_4_ aqueous solution at 70 °C for 1 h. The compact TiO_2_ layer (≈40 nm) used as the electron transport layer was fabricated after annealing the above FTO/TiO_2_ substrate at 200 °C for 30 min in air. The pristine perovskite precursor solution without the additive was prepared by dissolving FAI, MAI, and PbI_2_ in 1 mL DMF/DMSO (v/v 4:1), with a molar ratio of 0.85:0.15:1. Subsequently, different amounts of the DIFA additive (e.g., molar ratios of 1%, 2%, 3%, 6%, and 8% with respect to the PbI_2_ in the pristine precursor solution) were added. The perovskite films (≈450 nm) were prepared by spin‐coating technique and then annealed at 150 °C for 30 min. Spiro‐OMeTAD layer as hole transporting layer (HTL) was prepared by spin‐coating the related precursor onto the perovskite films. A 60 nm thick gold electrode was finally thermally evaporated onto the HTL‐coated film.

*Instruments and Characterization*: Surface morphology and cross‐section images were captured by a field‐emission scanning electron microscope (JEOL SU‐8020) equipped with energy‐dispersive X‐ray spectroscopy (EDS). Water contact angle measurement was performed on an OCA20 instrument. NMR spectra were recorded on a JEOL JNM‐ECZ 400S/L1 spectrometer. Mass spectrum was obtained on a MAXIS LC‐MS spectrometer. The conductivity can be determined by the slope of the *I–V* curve obtained from an electrochemical workstation utilizing a Au/perovskite/Au device under dark condition. The thickness of perovskite films can be measured by Stylus Profiler. Absorption spectra were obtained by a UV−vis spectrophotometer (Shimadzu UV‐3600). The steady state photoluminescence (PL) and time‐resolved photoluminescence (TRPL) spectra were measured using an Edinburgh Instruments Ltd. FLS980 spectrometer. Fourier transform infrared spectroscopy (FTIR) was performed on a Bruker EQUINX55 spectrometer. XRD patterns were carried out using a Bruker D8 GADDS Diffractometer with the Cu Kα radiation. The photocurrent density−voltage (*J−V*) curve of the PSC was obtained by using a Keithley Model 2400 digital source meter under an illumination of an AM 1.5 solar simulator (100 mW cm^−2^, SAN‐EI, Enlitech) with a scan rate of 30 mV S^−1^. The EQE spectra of the PSCs were measured using a QTest Station 500TI monochromator. EIS were recorded under dark condition with a frequency range from 4 × 10^6^ to 10 Hz.

## Conflict of Interest

The authors declare no conflict of interest.

## Supporting information

Supplementary
