# Supplementary material for: Additive Engineering to Grow Micron‐Sized Grains for Stable High Efficiency Perovskite Solar Cells
Source: Adv Sci (Weinh). 2019 Jul 26;6(18):1901241. doi: 10.1002/advs.201901241 (PMC6755530; doi:10.1002/advs.201901241)
Supplement: Supplementary file 1 — Supplementary [file ADVS-6-1901241-s001.pdf]

Copyright WILEY-VCH Verlag GmbH & Co. KGaA, 69469 Weinheim, Germany, 2019.

ADVANCED SCIENCE
Open Access

Supporting Information

for *Adv. Sci.,* DOI: 10.1002/advs.201901241

Additive Engineering to Grow Micron-Sized Grains for Stable High Efficiency Perovskite Solar Cells

Hua Li, Guohua Wu, Wanyi Li, Yaohong Zhang,* Zhike Liu, Dapeng Wang,* and Shengzhong (Frank) Liu**

Supporting Information

Additive engineering to grow micron-sized grains for stable high efficiency perovskite solar cells

Hua Li[a], Guohua Wu[a], Wanyi Li[a], Yaohong Zhang[b]*, Zhike Liu[a], Dapeng Wang[a]*, and Shengzhong (Frank) Liu[a]**

[a] Key Laboratory of Applied Surface and Colloid Chemistry, National Ministry of Education, Shaanxi Key Laboratory for Advanced Energy Devices, Shaanxi Engineering Laboratory for Advanced Energy Technology, School of Materials Science and Engineering, Shaanxi Normal University, Xi'an 710119, China.

[b] Faculty of Informatics and Engineering, The University of Electro-Communications, 1-5-1 Chofugaoka, Chofu, Tokyo 182-8585, Japan.

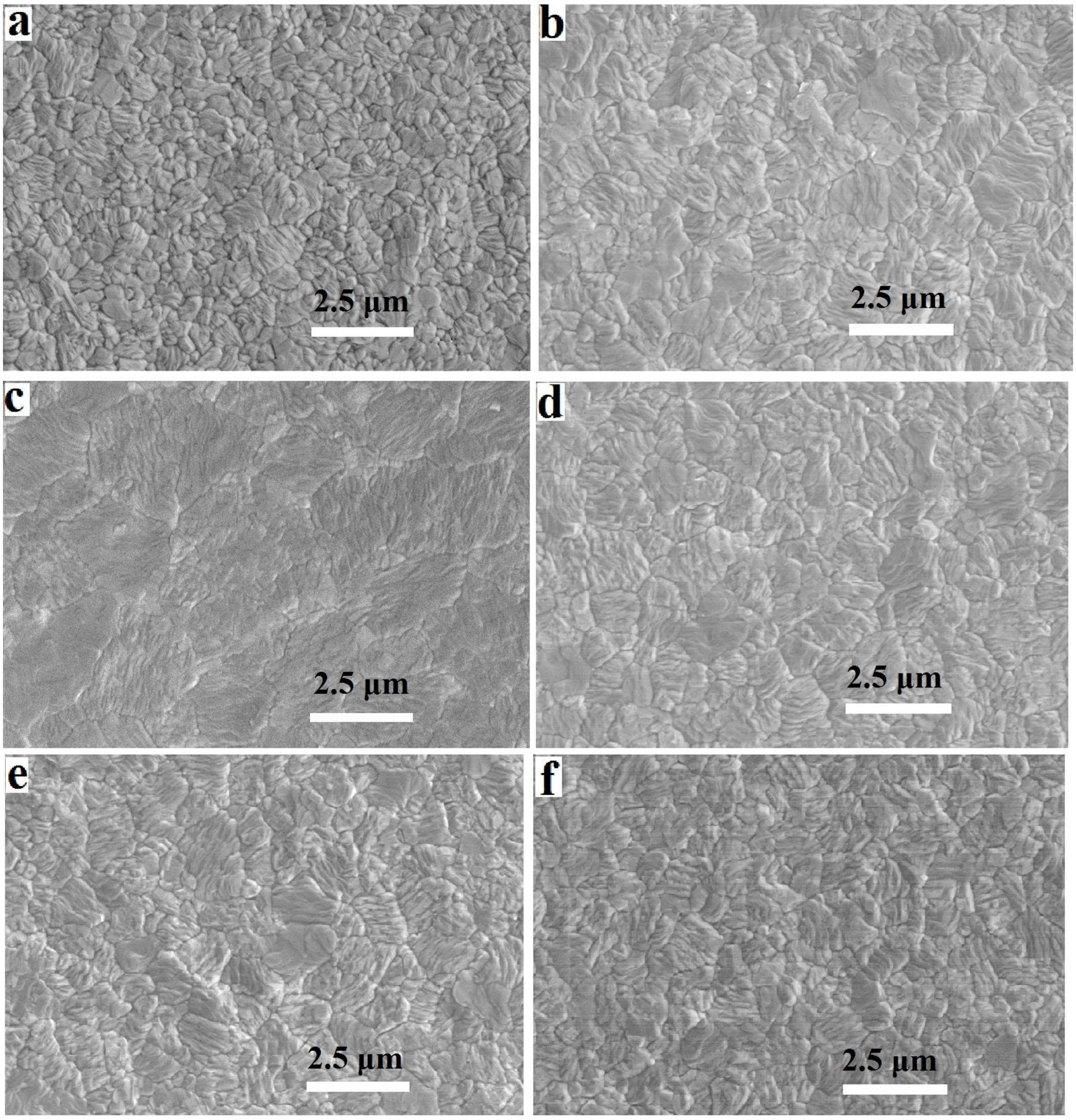

Figure S1. Surface-view scanning electron microscopy (SEM) images of (a) perovskite films without DIFA additive and with different molar ratios of (b) 1%, (c) 2%, (d) 3%, (e) 6%, and (f) 8% DIFA additive with respect to the PbI_2 in the pristine precursor solution.

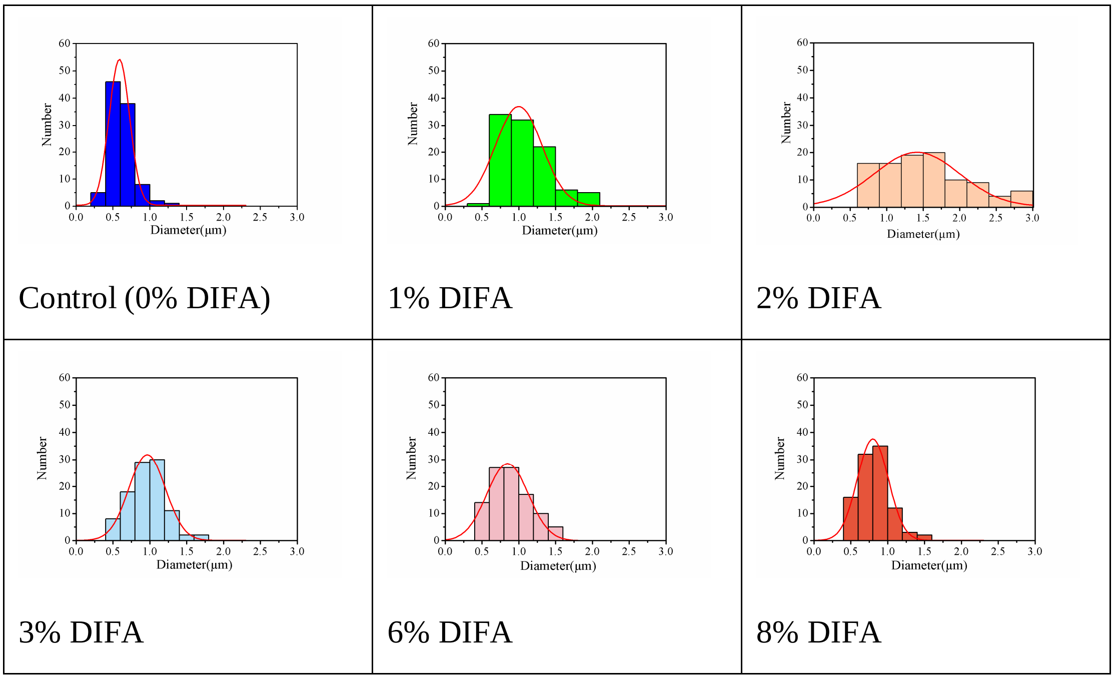

Figure S2. The distribution of perovskite crystal grain size with different concentrations of DIFA.

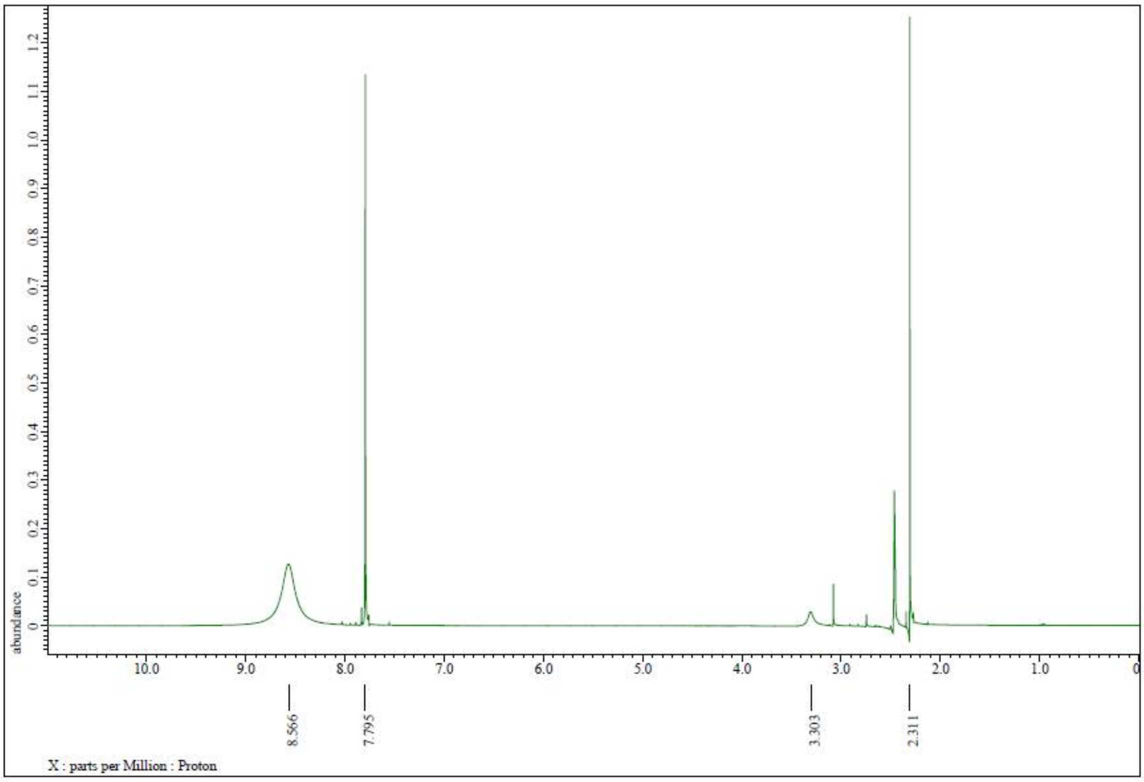

Figure S3. The ^{1}H NMR of $FA_{0.85}MA_{0.15}PbI_3$ perovskite precursor solution.

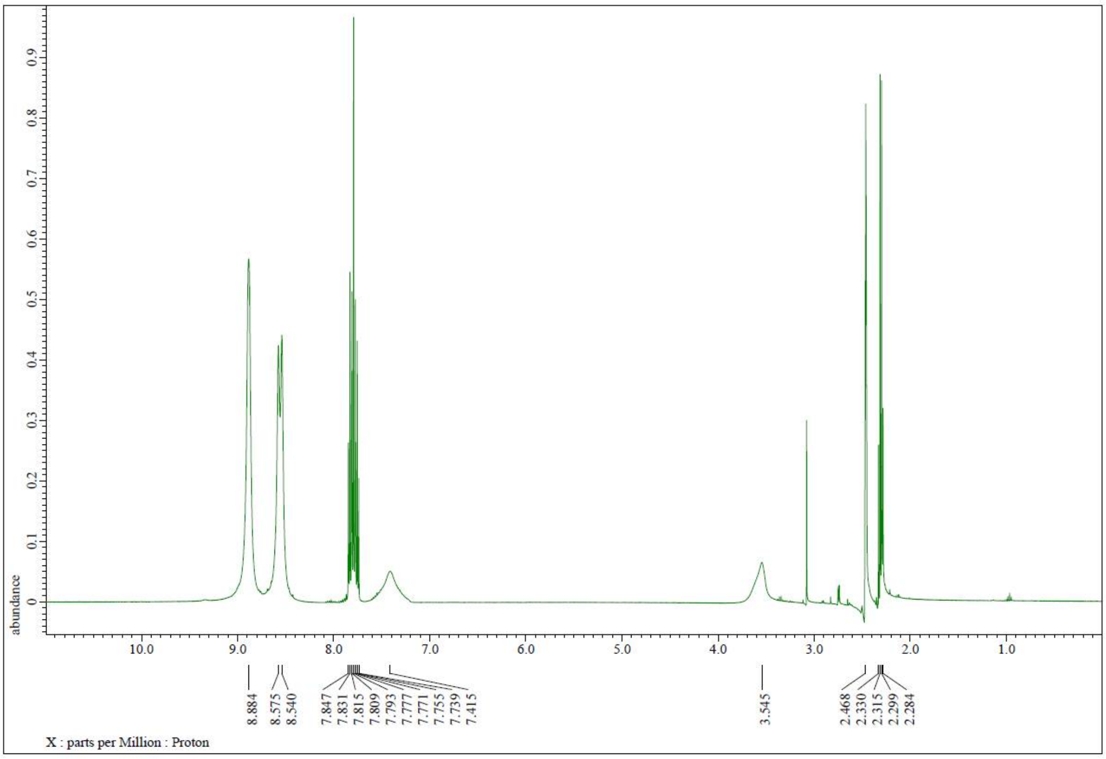

Figure S4. The ^{1}H NMR of $FA_{0.85}MA_{0.15}PbI_3$ perovskite precursor solution with 2% DIFA.

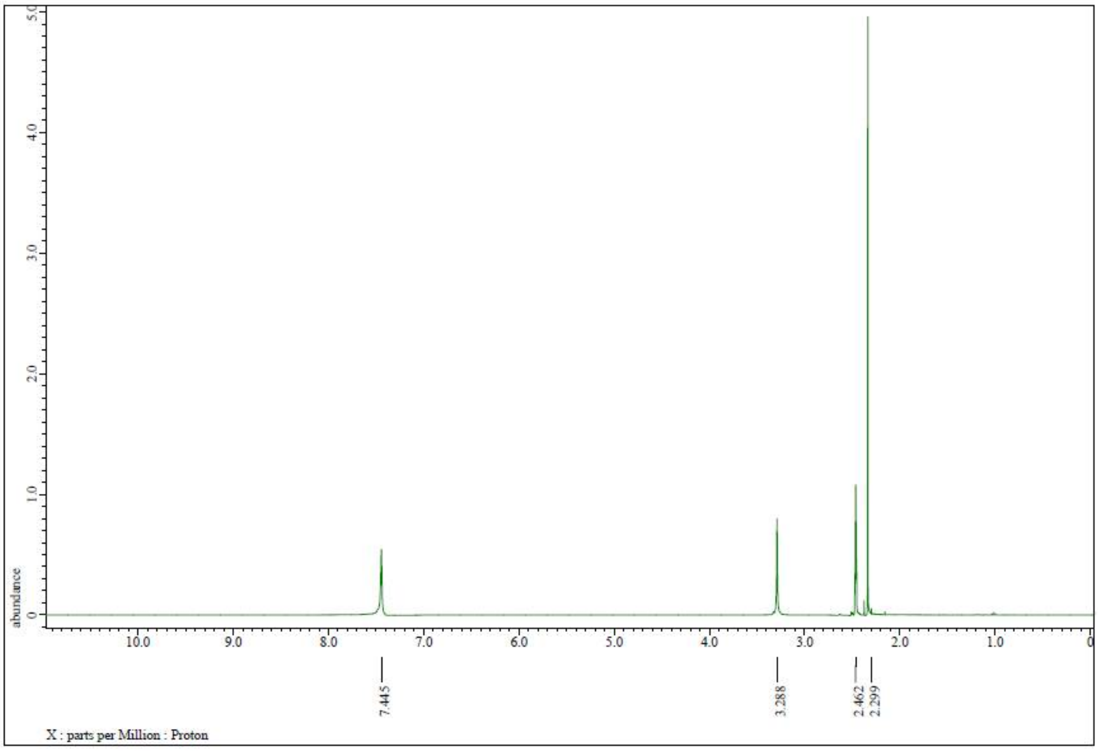

Figure S5. The ^{1}H NMR of $MAPbI_3$ perovskite solution.

Table S1. The average water contact angles of perovskite films with different concentrations of DIFA.

Sample	Contact angle (°)
Control	63.20±1.40
1% DIFA	73.30±1.08
2% DIFA	77.42±1.18
3% DIFA	78.05+0.95
6% DIFA	78.90±0.69
8% DIFA	80.02±0.58

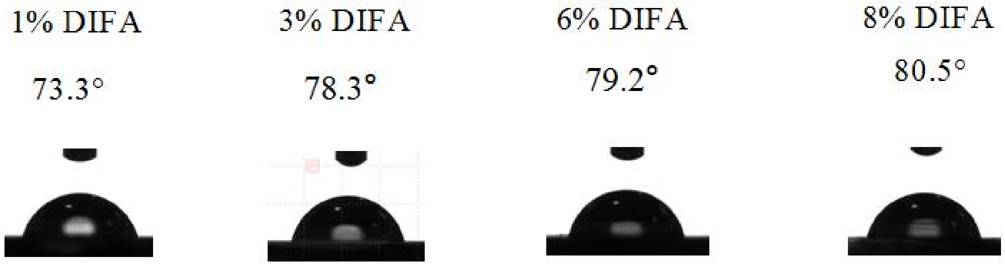

Figure S6. The water contact angles of perovskite films with different concentrations of DIFA.

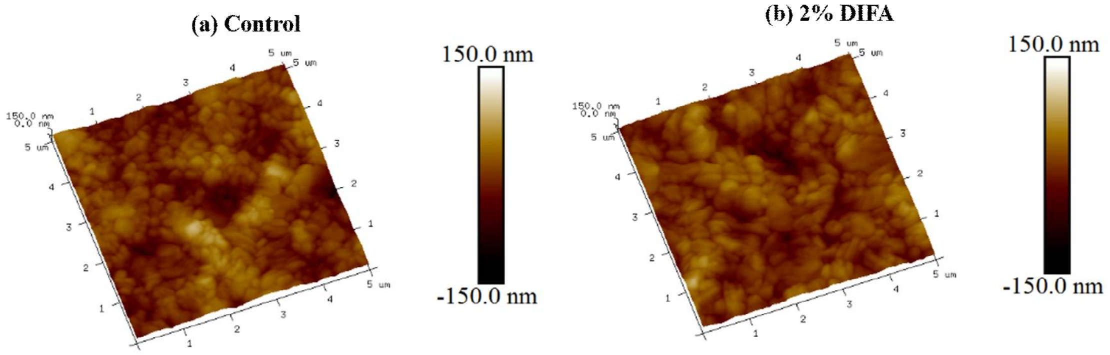

Figure S7. The AFM images of perovskite films on TiO_2 layer without (a) and with 2% DIFA (b).

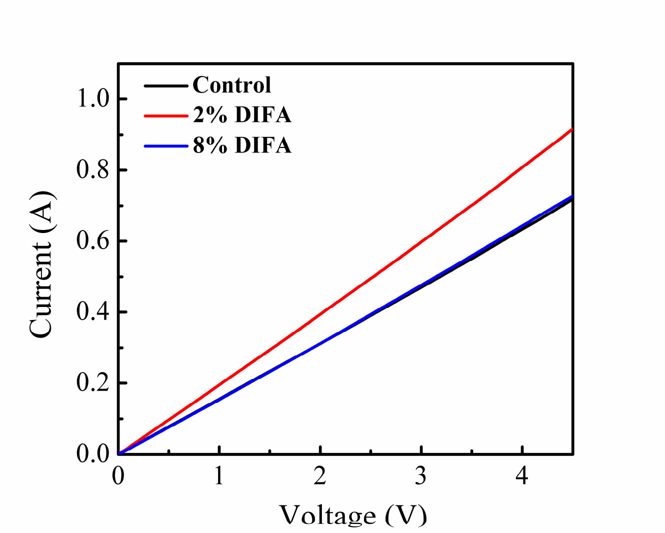

Figure S8. The *I-V* curves of perovskite films with 0%, 2%, and 8% DIFA under dark condition. The calculated conductivity is 7.12 × 10^{-3} S·cm^{-1} for pristine perovskite film, 9.89 × 10^{-3} S·cm^{-1} for 2% modified perovskite film, and 7.91 × 10^{-3} S·cm^{-1} for 8% modified perovskite film, respectively.

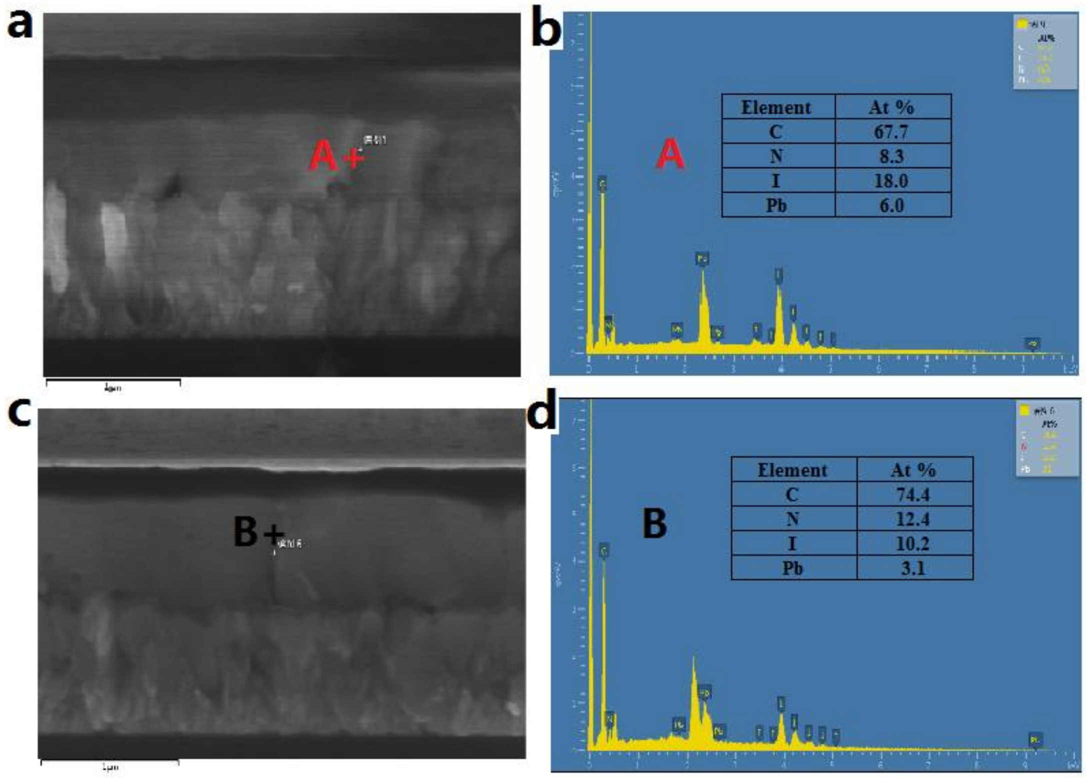

Figure S9. The SEM image of perovskite film without (a) and with (c) DIFA additive. (b) and (d) are the EDX spectra of point A and B in (a) and (c), respectively.

Table S2. Summary of fitting parameters of time-resolved PL spectra based on samples with different contents of DIFA.

Sample	τ_1 (ns)	Amplitude τ_1 (%)	τ_2 (ns)	Amplitude τ_2 (%)	τ_{ave} (ns)
control	34.15	7.99	6.71	92.01	15.12
1% DIFA	76.17	17.60	11.18	82.40	49.70
2% DIFA	79.68	24.52	16.05	75.48	55.33
3% DIFA	67.79	16.85	10.15	83.14	43.30
6% DIFA	55.18	14.58	8.53	85.42	33.01
8% DIFA	46.52	10.48	6.29	89.52	24.96

The time-resolved photoluminescence (TRPL) spectra can be fitted with a following biexponential equation [ref 1]:

$$f(t) = \sum_i A_i\, exp(-t/\tau_i) + B$$

where τ_i, A_i, and B are the decay time, decay amplitude, and a constant, respectively. As listed in Table S2, the decay time τ_1 in slow decay process is originated from the direct recombination of free carriers. The decay time τ_2 in the fast decay process is originated from the Shockley−Read−Hall (SRH) recombination. The lifetimes of 2% DIFA perovskite film (τ_1= 79.68 ns, τ_2 = 16.05 ns) are larger compared to those of the pristine perovskite film (τ_1 = 34.15 ns, τ_2 = 6.71 ns), which indicated the simultaneous

decrease in the recombination center and the defect state density in 2% DIFA perovskite film.

For conveniently comparison, the average PL decay times (τ_{av}) is proposed, which can be calculated according to the fitted A_i and τ_i values by the following equation:

$$\tau_{av} = \frac{\sum A_i \tau_i^2}{\sum A_i \tau_i}$$

The lifetime of 2% DIFA perovskite film (τ_{av}= 55.33 ns) is significantly larger compared to that of the pristine perovskite film (τ_{av} = 15.12 ns). These results confirmed that the perovskite film quality can be improved after the addition of 2% DIFA.

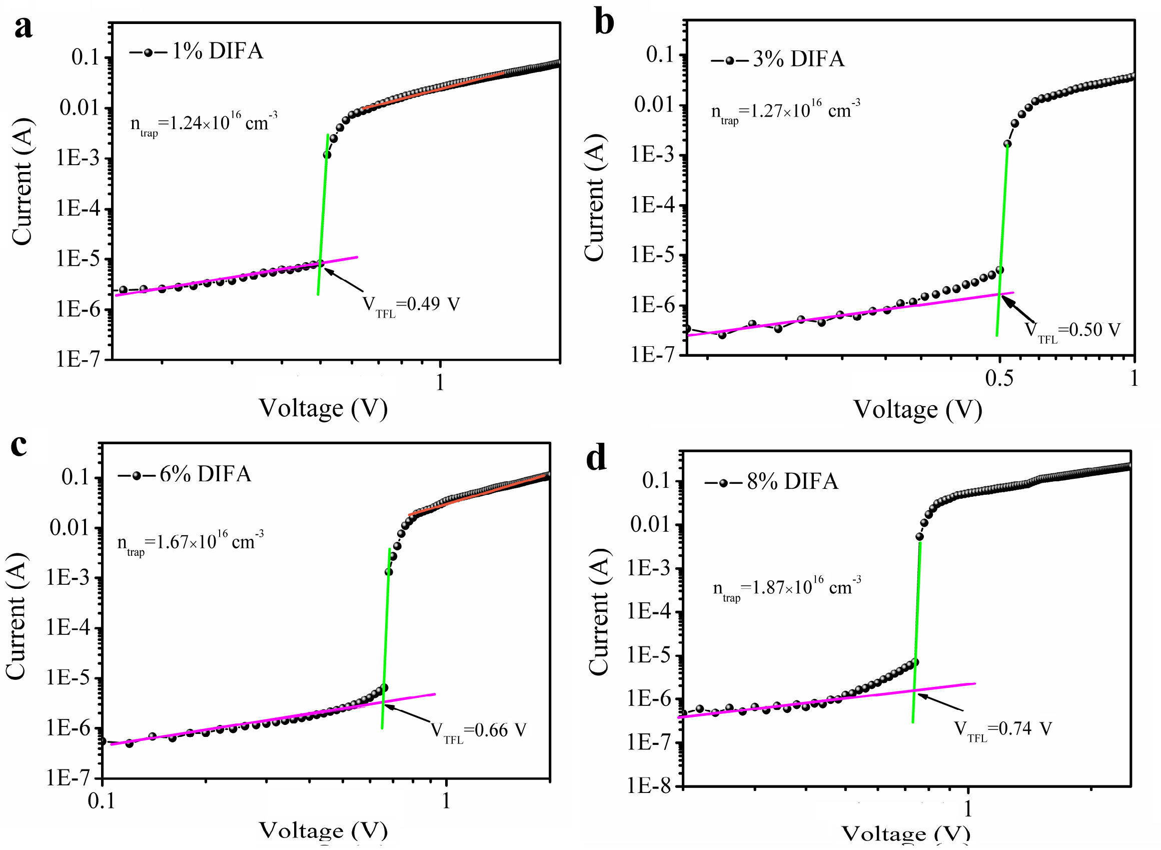

Figure S10. Dark current–voltage characteristics of electron only devices based on perovskite film with 1% (a), 3% (b), 6% (c), and 8% (d) DIFA.

Table S3. Summary of average photovoltaic parameters of the PSCs with different contents of DIFA under reverse scans.

PSCs	*V*oc (V)	*J*sc (mA/cm^2)	FF (%)	PCE (%)
Control	1.06±0.02	24.37±0.36	71.9±1.3	18.48±0.28
1%	1.07±0.02	24.67±0.28	74.7±1.3	19.73±0.56
2%	1.09±0.01	24.74±0.33	76.0±1.1	20.44±0.45
3%	1.08±0.01	24.63±0.27	74.5±1.4	19.75±0.54
6%	1.06±0.01	24.68±0.22	73.1±0.9	19.21±0.30
8%	1.07±0.01	24.41±0.41	72.6±1.7	18.95±0.33

Table S4. Summary of photovoltaic parameters of the PSCs with different contents of DIFA under reverse and forward voltage scans.

PSCs	Scan direction	*V*oc (V)	*J*sc (mA/cm^2)	FF (%)	PCE (%)	Hysteresis Index (%)
Control	Reverse	1.05	24.48	73.3	18.91	12.7
	Forward	1.01	24.46	66.8	16.50	
1%	Reverse	1.10	24.80	76.9	20.96	6.9
DIFA	Forward	1.06	24.73	74.2	19.52	
2%	Reverse	1.10	24.79	76.9	21.04	4.9
DIFA	Forward	1.07	25.04	74.7	20.00	
3%	Reverse	1.10	24.78	75.2	20.50	5.6
DIFA	Forward	1.07	24.75	73.1	19.35	
6%	Reverse	1.07	24.32	76.3	19.83	5.6
DIFA	Forward	1.04	24.30	73.8	18.72	
8%	Reverse	1.07	24.36	75.0	19.53	5.9
DIFA	Forward	1.04	24.31	72.4	18.38	

Table S5. EIS parameters for PSCs without and with 2% DIFA.

Device	*Rs* (Ω)	*Rrec* (Ω)	$C\mu$ (F)	τ_e (μs)
Control	18.00	238	1.152×10^{-8}	2.74
2% DIFA	12.60	500	8.980×10^{-9}	4.49

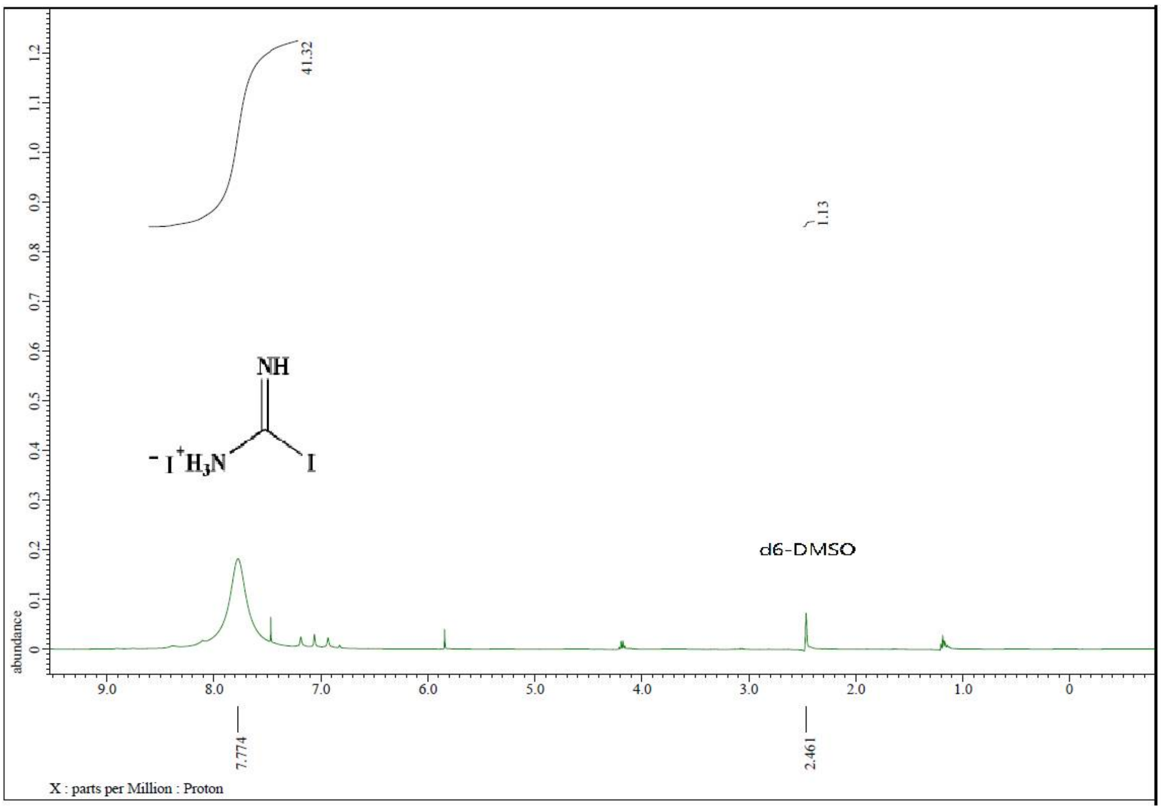

Figure S11. ^{1}H NMR of DIFA.

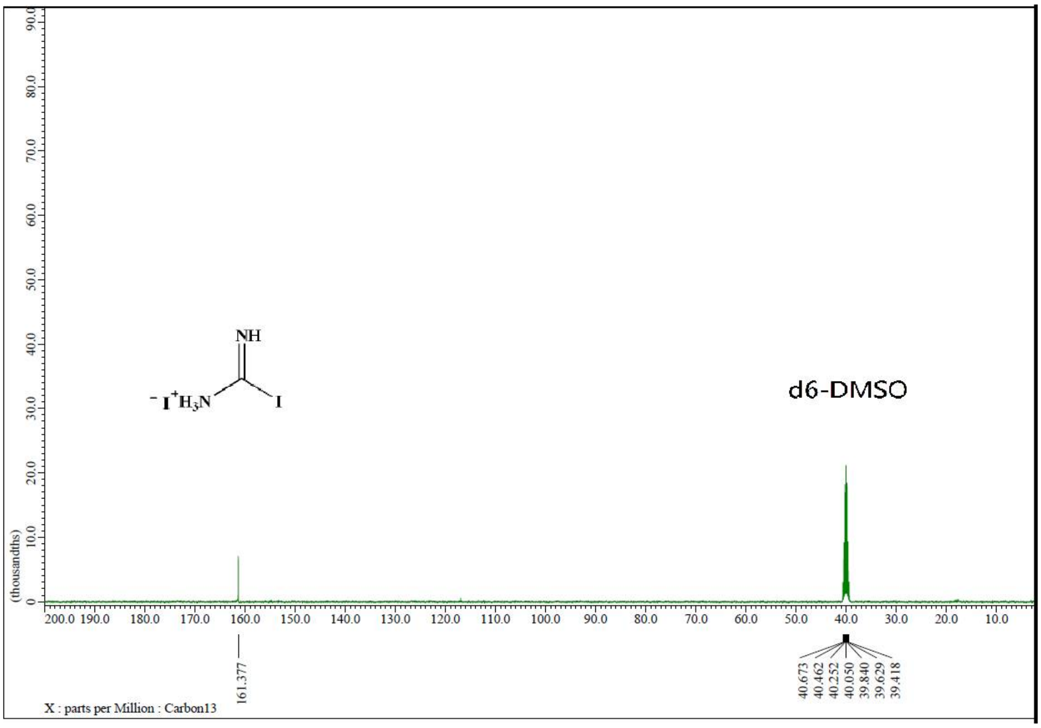

Figure S12. ^{13}C NMR of DIFA.

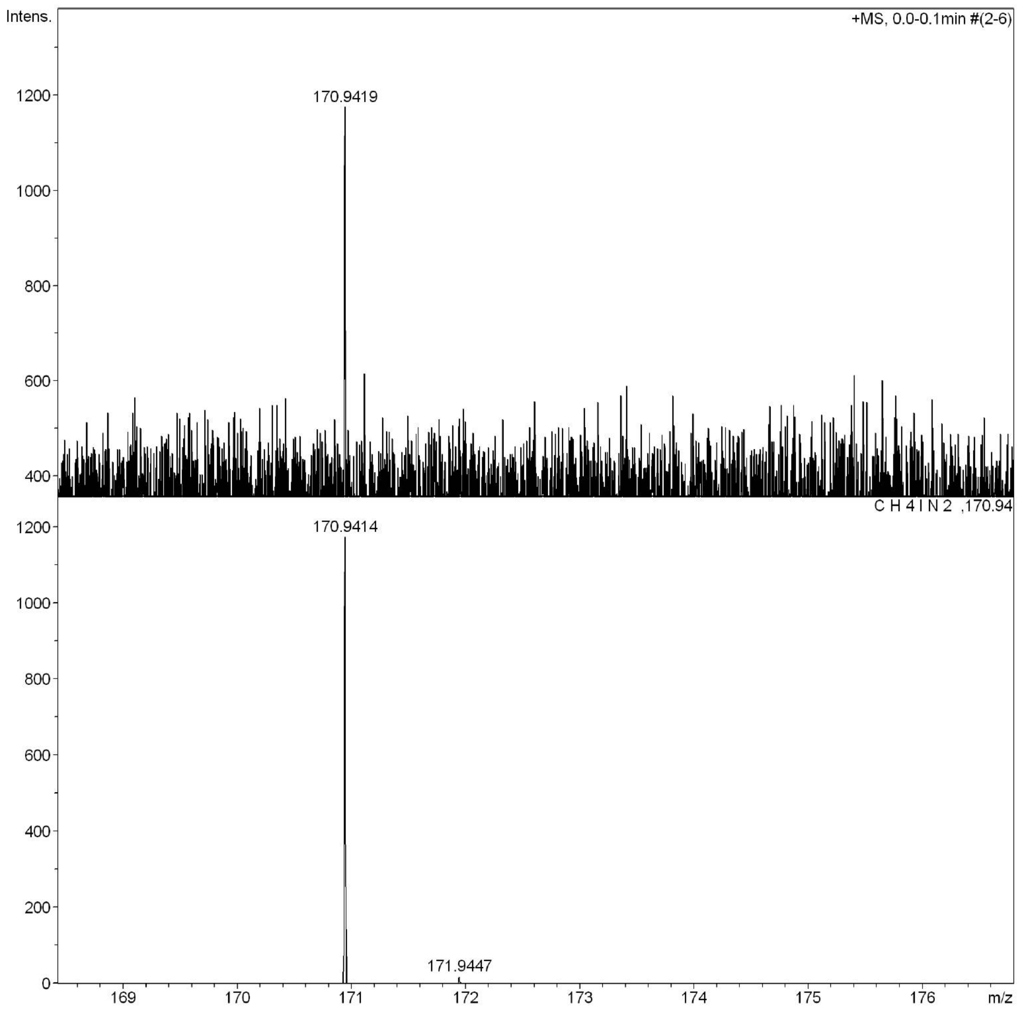

Figure S13. Mass spectrum of DIFA.

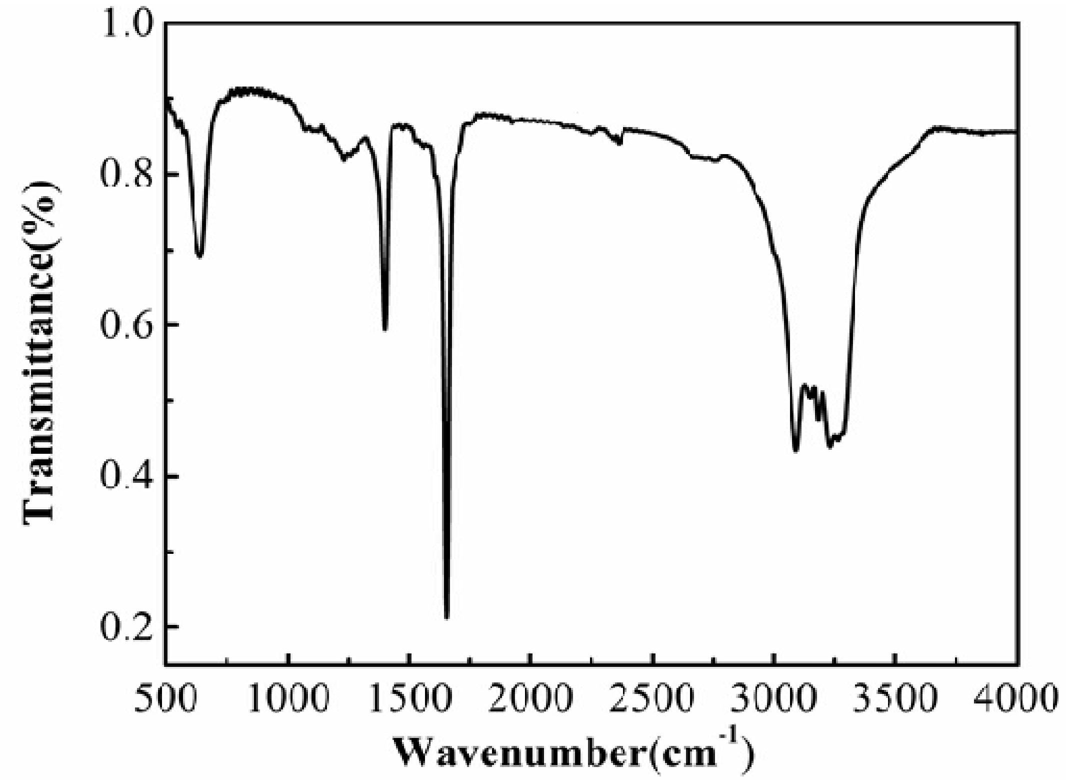

Figure S14. FT-IR of DIFA.

Ref 1: Jiang Hong, Yan Zhe, Zhao Huan, Yuan Shihao, Yang Zhou, Li Juan, et al., Bifunctional Hydroxylamine Hydrochloride Incorporated Perovskite Films for Efficient and Stable Planar Perovskite Solar Cells, ACS Applied Energy Materials, **2018**, 1(2):900-909.
